# Lifetime ostracism experiences and mechanisms of pain

**DOI:** 10.3389/fpain.2022.1037472

**Published:** 2022-12-16

**Authors:** Kaitlyn T. Walsh, Brandon L. Boring, Namrata Nanavaty, Adrienne R. Carter-Sowell, Vani A. Mathur

**Affiliations:** ^1^ Department of Psychological and Brain Sciences, Texas A&M University, College Station, TX, United States; ^2^ Department of Behavioral Health, Houston Methodist Hospital, Houston, TX, United States; ^3^ Department of Psychology, University of Oklahoma, Norman, OK, United States; ^4^ Diversity Science Research Cluster, Texas A&M University, College Station, TX, United States; ^5^ Texas A&M Institute for Neuroscience, Texas A&M University, College Station, TX, United States

**Keywords:** inequity, disparity, quantitative sensory testing, social exclusion, social indicators

## Abstract

One social mechanism by which marginalization is enacted is via ostracism. Recent research has demonstrated ostracism's impact on physical health, but little is known about the relationship between accumulated lifetime experiences of ostracism and pain. Despite recent calls for added attention to social modulation of pain and social indicators of pain disparities, the impact of specific social factors on pain—including those of ostracism—are not well understood. Results of laboratory studies on the effects of acute ostracism experiences on pain sensitivity have been mixed. However, these studies have not considered lived and repeated experiences of ostracism, and primarily included single static measures of pain sensitivity. Additionally, inclusion and representation of the relationship between ostracism experiences and pain among people with minoritized identities are lacking in the current literature. In this study, we explored accumulated lifetime experiences of ostracism as a potential contributing factor to enhanced pain and one social mechanism by which societal inequity may create and maintain inequity in pain. We extracted measures of lifetime experiences of ostracism from six studies focused on social factors and (non-chronic) pain conducted between 2016 and 2020 (*n* = 505 adults). To retain and examine diversity within the sample, we used moderation and within-group analyses. Results indicate that greater experiences of lifetime ostracism are associated with lower cold pain tolerance, but not other pain measures, in the whole sample. Moderation and within-group analyses reveal opposing patterns of results between populations included in the extant literature (White participants, convenience samples) and those under-represented in the scientific literature (racialized groups, community samples). This study provides an example of a diversity science approach to examining social indicators of pain, illustrates the limited generalizability of previous studies on ostracism and pain, and highlights the need for increased representation and inclusion to understand mechanisms of pain and inequity.

## 1. Introduction

Minoritized communities experience a disproportionate burden of illness and disease. These health disparities are driven by systemic inequality embedded within institutions that serve to marginalize and disadvantage groups across socially constructed demographic lines (1, 2). One social mechanism that serves to marginalize others and has demonstrated impact on health is social ostracism. Ostracism, or the experience of being ignored or socially excluded by others, is common, and is experienced more frequently by individuals and communities with stigmatized and/or marginalized identities (3, 4). The impact of ostracism on mental health is well-established: those who have greater experiences of ostracism show greater levels of anxiety and depression, as well as lower self-reports of psychological well-being (5–7). Recent studies demonstrate the impact of ostracism—and related concepts such as social isolation and loneliness—on physical health both acutely [e.g., greater physiological responses such as increased blood pressure and inflammatory reactivity (8)], and chronically [e.g., development of chronic diseases such as coronary heart disease and stroke, as well as all-cause mortality (9–11)]. However, despite recent calls for greater attention to social modulators of pain (12, 13) and social indicators of pain disparities (14–16), several important social factors—including ostracism—have been understudied in relation to pain.

Constructs related to ostracism—such as social isolation and loneliness—predict worse pain outcomes such as greater pain interference and disability among individuals with chronic pain (17–22). Similar patterns may be expected for ostracism; however, ostracism critically differs from loneliness and social isolation in that it involves the directed action of others (i.e., being ignored or excluded by another person or group of people), and thus may be more systematically distributed to stigmatized groups or individuals than loneliness or isolation.

The few studies that have examined ostracism in the context of pain have used experimental paradigms and have yielded mixed results. Some research has found that experiencing ostracism *facilitates* pain as evidenced by increased or hypersensitivity to acute pain stimuli (23), while other studies found ostracism to have a *numbing* effect evidenced by decreased or hyposensitivity to pain (24, 25). Findings from Bernstein and Claypool (26) suggest that these mixed findings may be due to methodological differences in how feelings of ostracism were manipulated. In their study, Bernstein and Claypool used two different manipulations of ostracism that reflect either an acute, less severe level of ostracism (e.g., exclusion in an online ball-tossing game), or a chronic, more severe level of ostracism (e.g., results from a bogus personality test indicating a future of being alone). Less severe, acute levels of ostracism were associated with hypersensitivity while more severe levels of ostracism were associated with hyposensitivity to experimental pain stimuli. However, the previous studies investigating ostracism and physical pain have limited generalizability due to relying on experimentally manipulated measures of ostracism and simple or single measures of pain sensitivity (i.e., cold pain sensitivity).

The purpose of the current study was to examine the relationship between real-lived experiences of ostracism and laboratory tests of pain sensitivity. We hypothesized that greater lived experiences of ostracism would be associated with increased pain sensitivity across multiple pain measures. In recognition that experiences of ostracism are systematically different among racialized groups, we also hypothesized that lived ostracism would be experienced more among members of racialized groups. Finally, to probe ostracism as a potential social indicator of pain inequity, we explored the relationship between lived ostracism and laboratory pain within racialized groups.

## 2. Materials and methods

### 2.1. Data source and extraction

Secondary data analysis was performed on data collected across six studies conducted between 2016 and 2020. Specifically, only measures of participant identity (demographics), lived ostracism, and laboratory pain were extracted. All studies were conducted using standardized training and study protocols, with the same equipment in the same laboratory—thus minimizing measurement variability and further enhancing power to detect small effects. Of the six studies, five relied on traditional forms of convenience sampling (four student samples recruited through student participant pools and one that recruited on campus and within the community using posted fliers) while one study recruited exclusively from the community (using fliers posted in local businesses and craigslist) and excluded individuals affiliated with the university. Four studies included experimental manipulations (random assignment to an experimental or control condition) and post-experimental pain assessment which could confound the impact of ostracism experiences on pain sensitivity, and thus for these studies, only participants in the neutral/non-experimental condition (*k* = 1) or only pre-manipulation baseline measures of pain (*k* = 3) are included in the present analysis. All studies were approved by the Texas A&M University Institutional Review Board.

### 2.2. Participants

A total of 511 adults were enrolled and completed studies that included the ostracism experiences scale and any laboratory pain measure. Six participants were excluded from analyses: three because they did not complete the primary ostracism measure, two because they requested their data not be retained, and one because a participant disclosed disqualifying information after completing the study (i.e., they provided inaccurate responses during eligibility screening), resulting in a final analysis sample of 505 participants. Eligibility criteria included being at least 18 years old, no recent or current chronic or acute pain, and no use of pain medications within the past 3 days. Participation in any of these studies was an exclusion criterion for the others, ensuring the independence of samples. Depending on the study, participants received either course credit or monetary compensation at a rate of $12–$20/h for their time. Sample demographics are reported in Table 1. A subset of the participants (i.e., 120 Latinx Americans) from the current analysis were included in a separate, previously reported analysis on multidimensional racialized discrimination experiences (27).

**Table 1:** Sample descriptive characteristics by study.

	Sampling strategy						
	Convenience					Community	Total
Study #	1	2	3	4	5	6	
Sample size (*N*)	86	52	154	99	81	33	505
Mean age (*SD*)	19.01 (1.15)	18.92 (0.93)	19.19 (2.18)	18.90 (1.14)	21.95 (4.25)	30.50 (10.19)	20.24 (4.43)
**Sex**							
*Female*	49	34	122	68	41	15	329
*Male*	37	17	32	31	40	17	174
*Unspecified*	0	1	0	0	0	0	1
*Missing*	0	0	0	0	0	1	1
**Racialized identity**							
*Asian/Asian American*	3	1	9	23	2	0	38
*Black/African American*	5	6	4	10	26	6	57
*Hispanic/Latinx American*	29	16	42	41	32	3	163
*Multiracial*	1	2	9	10	0	1	23
*Native American/Alaskan native*	1	0	0	3	0	0	4
*White/European American*	45	27	89	12	21	21	215
*Middle Eastern*	1	0	1	0	0	0	2
*Lebanese*	1	0	0	0	0	0	1
*Afro-dominican*	0	0	0	0	0	1	1
*Missing*	0	0	0	0	0	1	1

### 2.3. Procedure

Prior to any study procedures, all participants were screened for study eligibility and completed a detailed in-person informed consent process. The specific laboratory pain measures included in analysis per study are reported in Table 2. As part of the standardized laboratory pain protocols implemented across studies, breaks were implemented between pain measures in order to avoid sensitization. All questionnaires were electronically administered using Qualtrics (Provo, UT).

**Table 2:** Procedure order by study.

	Procedure order
Study 1	MTS, OES
Study 2	EXP, CPTh/Tol^sec^, CPInt^removal^, CPInt^max^, AS, OES
Study 3	CPTh/Tol^sec^, CPInt^removal^, EXP, EXPpain, OES
Study 4	OES (completed prior to laboratory visit), MTS (128 mN), EXP, EXPpain
Study 5	Randomized [QST1 (CPTh^°C^, HPTh/Tol^°C^, PPTh^kPa^), QST2 (MTS, CPM x2)], OES
Study 6	PPTh^kPa^, CPTh^°C^, HPTh/Tol^°C^, MTS, CPM, CPTh/Tol^sec^, CPInt^removal^, CPInt^max^, AS, OES, EXP, EXPpain

MTS, temporal summation of mechanical pain using weighted pinprick stimulators (middle finger); OES, administration of questionnaires including the Ostracism Experiences Scale; EXP, experimental manipulation (only neutral/non-experimental control condition included in the present analysis); CPTh/Tol^sec^, cold pain threshold and tolerance (seconds) determined using cold water bath (left hand); CPInt^removal^, cold pain intensity when left hand was removed from the cold water bath; CPInt^max^, self-reported maximum cold pain intensity while hand was in the cold water bath; AS, after sensations (intensity at 30 s and duration) after cold water bath procedure; EXPpain, post manipulation pain assessment not included in the present analysis; CPTh^°C^, cold pain threshold (degrees Celsius) determined using contact probe (right forearm); HPTh/Tol^°C^, heat pain threshold and tolerance (degrees Celsius) determined using contact probe (right forearm); PPTh^kPa^, pressure pain threshold (kilopascals) determined using pressure algometer (right trapezius); CPM, conditioned pain modulation [conditioning stimulus: cold water bath (left hand); test stimulus: pressure algometer (right trapezius)].

### 2.4. Materials

#### 2.4.1. Ostracism experiences scale

The Ostracism Experiences Scale (OES) is an 8-item self-report survey that measures the frequency and accumulation of an individual's real-lived ostracism experiences (28, 29). Examples of items include: “*In general, others keep me out-of-the-loop on information that is important to my close relationship*” and “*In general, others do not look at me when I’m in their presence*”. Participants rated the frequency at which they have these experiences on a 6-point Likert scale, ranging from 1 (Hardly Ever) to 7 (Almost Always). The items were summed to create a total score of real-lived ostracism experiences (*α* = 0.92, range across studies = 0.91–0.96), with higher scores indicating more ostracism.

#### 2.4.2. Demographics

Age, sex, and racialized identity were assessed across studies. Participants were asked “*What is your age*” and provided their age in years using an open text box. Participants were prompted to provide their self-reported sex and racialized identity using the following items: “*Sex: (select one)* or *Gender: (select one)* [Male, Female, Other (please specify)]” and “*Ethnicity: (select one)* [African American/Black, Asian/Asian American, Hispanic/Latinx American, Native American/Alaskan Native, White/European American, Multiracial, Other (please specify)]”. We acknowledge that gender identity, and not sex, is the most appropriate measure to examine gender inequities in pain. Problematically, one study conflated sex with gender by asking for “gender” but only providing self-identification options for sex. All but this one study assessed sex and gender identity with separate questions. To maximize the sample size for inferential analyses, and because gender differences were not the primary aim of the present study, we use the imperfect measure of sex which allowed for self-identification but lacked explicit inclusion of intersex individuals. It is also important to note that the use of “other, please specify” is not inclusive and may have discouraged responses outside of the binary. Similarly, more recent studies include additional questions to more specifically and inclusively assess racialized, ethnic, and cultural identity. We use the initial question asking about racialized identity since it was included in all studies. However, the labels used are not inclusive of all identities and there is substantial heterogeneity in the lived and social experiences within racialized groups—particularly for multiracial identities.

### 2.5. Laboratory pain measures

Laboratory pain measures followed standardized protocols that we implement across studies, have previously reported (e.g., (27, 30, 31)), and summarize below.

#### 2.5.1. Cold pain

##### 2.5.1.1. Cold pain threshold

Cold pain threshold (CPTh) was operationalized either as the temperature at which a stimulus first became painful (CPTh^°C^) or the elapsed time at which a cold stimulus first became painful (CPTh^sec^).

CPTh^°C^ was assessed using a 30 mm × 30 mm ATS Thermal Stimulator (Pathway; Medoc, Ramat Yishai, Israel; *k* = 2) applied to the volar side of the right forearm. For each trial, the thermode gradually decreased in temperature, from a baseline of 32°C at a rate of 1°C/s until the participant pressed a button when the stimulus first became painful. If the stimulus reached the temperature limit (0°C), participants were asked to rate their current pain (0 (no pain) to 100 (the worst pain imaginable)), and if they indicated the presence of pain (>0), participant instructions were clarified before the next trial. In the analysis stage, such trials (where participants appeared to exceed CPTh) were marked as invalid and removed from calculation.

A total of 3 trials were conducted and CPTh^°C^ was calculated as the average of the 3 trials.

CPTh^sec^ was obtained via submersion of the left hand up to the wrist in a 4°C circulating cold-water bath (*k* = 3; specifically a PD15R-30 Polyscience Circulating Bath (Niles, IL, U.S.A.) (*k* = 1) and a Thermo Firsher Scientific Circulating Bath (Newington, NH, U.S.A.) (*k* = 2). CPTh^sec^ was calculated as the number of seconds the participant kept their hand in the water until they first felt pain as indicated by saying the word “pain”.

##### 2.5.1.2. Cold pain tolerance

Cold pain tolerance (CPTol^sec^) was assessed during the same trials as CPTh^sec^, operationalized as the total number of seconds a participant kept their hand in the water, following instructions to keep their hand in the water until they could no longer tolerate the pain.

##### 2.5.1.3. Cold pain intensity

Immediately after they removed their hand from the cold-water bath, participants were prompted to verbally indicate their current level of pain after removing their hand from the water (CPInt^removal^; *k* = 3), as well as the maximum amount of pain they felt while their hand was still in the water (CPInt^max^; *k* = 2) using the 0–100 scale described above.

#### 2.5.2. Heat pain

##### 2.5.2.1. Heat pain threshold

Heat pain threshold (HPTh^°C^) assessment followed the same procedure as CPTh^°C^ (*k* = 2), except the temperature gradually *increased* from the 32°C baseline.

##### 2.5.2.2. Heat pain tolerance

Heat pain tolerance (HPTol^°C^) (*k* = 2) was collected after the HPTh^°C^ trials using the same equipment and procedure. The thermode gradually increased in temperature until the participant pressed a button when the pain from the heat became *intolerable*.

#### 2.5.3. Pressure pain

Pressure pain threshold (PPTh^kPa^) was collected (*k* = 2) using a single electronic algometer (Algometer Type II; SBMEDIC Electronics, Solna, Sweden). Pressure was applied to the trapezius muscle and increased steadily at a rate of 50 kPa/s until the participant verbally indicated when the pressure first produced a painful sensation by saying “pain”. PPTh^kPa^ was calculated by averaging the two closest values (kPa) out of all the administered trials. One study (*k* = 1) administered a maximum of 3 PPTh trials and 1 study continued assessment until the experimenters obtained 2 values within 50 kPa.

#### 2.5.4. Temporal summation

Mechanical temporal summation (MTS) was assessed (*k* = 4) using weighted punctuate probes with a flat contact area of 0.2 mm diameter, delivering stimuli at a rate of 60 Hz (1 touch/second) to the middle phalange of the middle finger. MTS was calculated as the difference in verbal pain ratings on a 0–100 scale between the series of 10 stimuli and the single stimulus. A 128 mN probe was always used, though some (*k* = 3) studies also included tests for MTS using 256 and 512 mN probes. Larger, positive differences between the single and repeated stimulus ratings indicate greater summation of pain.

#### 2.5.5. Conditioned pain modulation

Conditioned pain modulation (CPM) was assessed (*k* = 2) by combining the PPTh^kPa^ (test stimulus) and CPTol^sec^ (conditioning stimulus) procedures. The test stimulus was applied after 20 s of the conditioning stimulus (or earlier if necessitated by participant hand-removal). The procedure ended (i.e., both stimuli were removed) when participants verbally indicated that the test stimulus first produced pain (PPTh^kPa^). CPM was calculated as the difference between this assessment of PPTh^kPa^ and the assessment obtained earlier in the study (in the absence of a conditioning stimulus). Larger, positive values indicate a greater conditioned modulation of pain. One study included two trials for CPM. For this study, we calculated the CPM outcome variable for each trial separately and then averaged the two values to obtain a single CPM outcome variable.

#### 2.5.6. After sensations

After sensations were assessed after CPInt^max^ (*k* = 2) using the same 0–100 scale. After sensations were assessed at least every 30 s until the participant rated their pain a zero. After sensations were calculated in 2 ways across each study: (1) the duration of after sensations (AS^duration^)—operationalized as the last time point (number of seconds) in which the participant rated pain above a zero, and (2) the intensity of after sensations (AS^intensity^)—operationalized as the participants’ pain rating 30 s after hand-removal (this time point was selected as the first common time point across studies).

### 2.6. Analyses

Given the exploratory nature of this study, we report all comparisons without alpha adjustment [e.g., (32)]. Before conducting inferential statistics, all variables were examined for normality and outliers were identified using graphical plots. Lifetime ostracism scores and laboratory pain measures were transformed—selecting the transformation that most effectively reduced the amount of skew—for inferential statistical analyses [e.g., (33)]. Before applying logarithmic transformations to variables containing negative or zero values, constants were added so that the minimum value was equal to 1 and all data points were included in statistical analyses. Bivariate correlations were conducted, and corresponding scatterplots inspected, to determine the relationship between ostracism experiences with laboratory pain.

Our approach to statistical considerations related to participant-level variables was informed by diversity science and anti-racism approaches—aiming to represent as opposed to control for diversity in lived experiences. Thus, we used moderation to examine potential within-group relationships in this study. Specifically, we first probed potential effects of racialized identity, sex, and age on the relationship between ostracism experiences and laboratory pain through moderation analyses and visual inspection of graphical plots. Moderation by study was also probed to detect potential design-related or history effects. Significant moderation was followed up with conditioning (i.e., the Johnson-Neyman technique) for continuous moderators and with within-group analyses for categorical moderators.

### 2.7. Missing data

All available data are included in analyses. In cases where there are fewer observations (sample size) for a given test, this was primarily due to the individual/primary study design (i.e., not all studies included all laboratory pain measures) as well as inclusion criteria for the present analysis (i.e., laboratory pain measures collected after an experimental manipulation were not extracted/included). Participants were also permitted to skip or stop any procedure at any time, though this was not common. We report the total sample used for each inferential analyses in the results section.

## 3. Results

### 3.1. Descriptive statistics

Participants were on average 20.24 (*SD* = 4.43) years of age and primarily identified as female (65.1% identified as female, 34.5% as male, 0.2% did not identify as male or female but did not provide further self-identification, and 0.2% did not provide an answer). Studies aimed to recruit a diverse sample of racialized identities with 7.5% of participants identifying as Asian or Asian American, 11.3% as Black or African American, 32.3% as Latinx or Hispanic American, 4.6% as Multiracial, 42.6% as White, and <1% (*n* < 5) as each of the following: Native American/Alaska Native, Middle Eastern, Lebanese, and Afro-Dominican; 0.2% did not disclose their racialized or ethnic identification.

Descriptive statistics for the primary predictor and outcome variables are reported in Table 3. The majority of ostracism scores fell below the scale midpoint (midpoint = 28; *M* = 15.72, *SD* = 8.43). Although the full range of possible scores was observed in this sample, 14.7% of participants reported “*Hardly Ever*” experiencing ostracism as indicated by the lowest possible total score on the OES (minimum score = 8) while only 0.2% reported “*Almost Always*” experiencing ostracism (maximum score = 56).

**Table 3:** Descriptive characteristics of lifetime ostracism and laboratory pain measures.

Variables	*N*	Mean	SD	Min	Max
Lifetime ostracism (OES)	505	15.72	8.43	8	56
**Laboratory pain**					
**Thresholds**					
*Pressure pain threshold (kPa)*	114	350.21	161.79	109	867
*Cold pain threshold (seconds)*	235	11.87	8.44	1.06	47.1
*Cold pain threshold (°C)*	113	10.69	7.76	0	26.73
*Heat pain threshold (°C)*	114	41.89	3.39	34.37	49.67
**Suprathreshold pain**					
*Cold pain tolerance (seconds)*	237	53.7	67.36	7.42	300
*Cold pain intensity (removal of stimulus)*	239	50.2	24.6	0	100
*Cold pain intensity (retrospective maximum)*	85	65.62	22.07	12	100
*Heat pain tolerance (°C)*	114	46.53	2.53	39.6	51.07
**Temporal summation**					
*128mN Stimulus*	298	6.31	9.05	−15	58
*256mN Stimulus*	199	10.24	11.74	−5	79
*512mN Stimulus*	198	14.87	14.25	0	70
**After sensations**					
*Pain severity rating at 30 s*	85	29.71	21.22	0	85
*After sensation duration (seconds)*	85	112.59	88.25	15	330
**Conditioned pain modulation**	114	109.71	138.13	−469.5	775.5

### 3.2. Bivariate correlations

Lifetime experiences of ostracism were not associated with age or sex and means did not differ across studies (*p* > 0.05). Greater lifetime ostracism experiences were significantly associated with lower CPTol^sec^ [*r*(237) = −0.149, *p* = 0.022]. No other simple relationships were statistically significant (0.018 < *r* < 0.140, 0.106 < *p* < 0.812).

### 3.3. Moderation

#### 3.3.1. Sex

**Figure 1:**
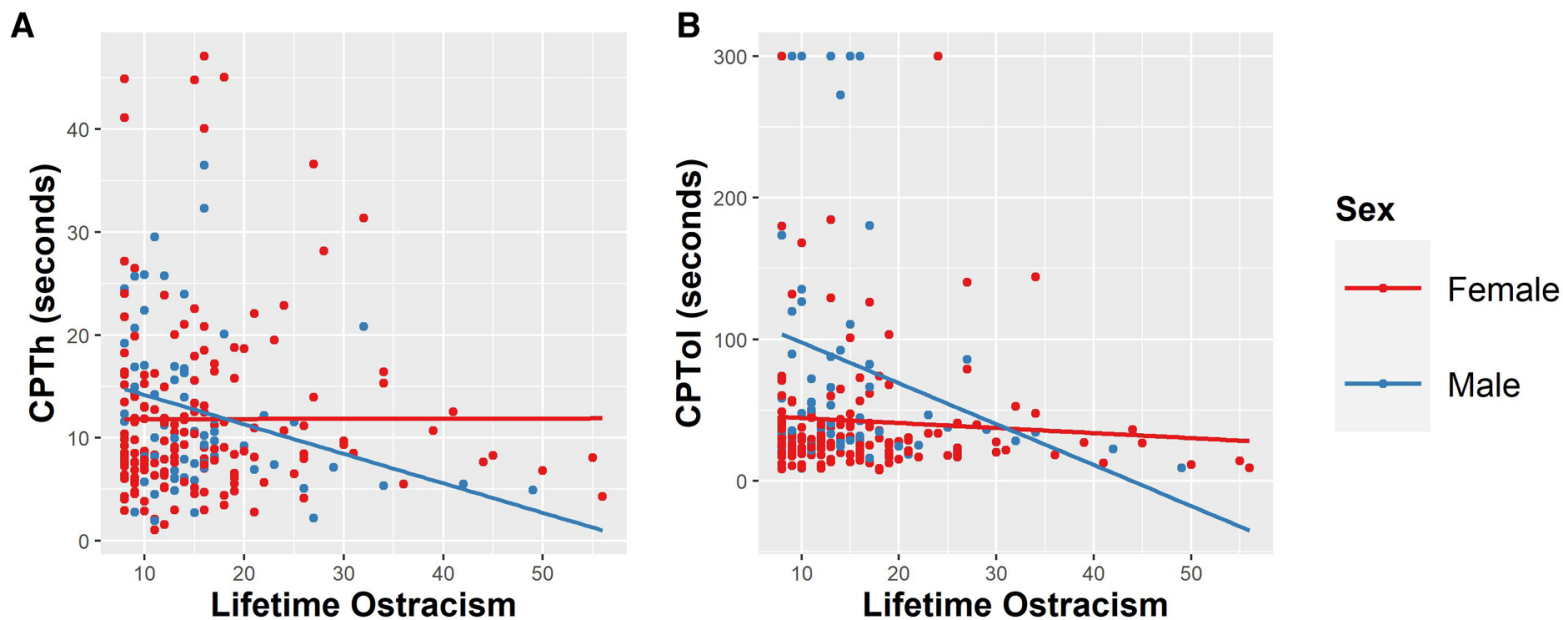
Sex significantly (*p* < 0.05) moderated the relationship between lifetime ostracism experiences and (**A**) cold pain threshold (CPTh^sec^) and (**B**) cold pain tolerance (CPTol^sec^). The raw, untransformed values are presented in the figure.

Sex significantly moderated the relationship between lifetime ostracism experiences and CPTh^sec^ [ΔR^2^ = 0.024, *F*(1, 229) = 5.686, *p* = 0.018] and CPTol^sec^ [ΔR^2^ = 0.020, *F*(1, 231) = 5.437, *p* = 0.021], such that the relationship was significant among male but not female participants. For male participants, greater lifetime ostracism experiences were associated with lower CPTh^sec^ [*r*(65) = −0.288, *p* = 0.020] and CPTol^sec^ [*r*(66) = −0.332, *p* = 0.007; Figure 1].

#### 3.3.2. Age

Age did not significantly moderate the relationship between lifetime ostracism experiences and laboratory pain (*p* > 0.10).

#### 3.3.3. Study selection strategy

**Figure 2:**
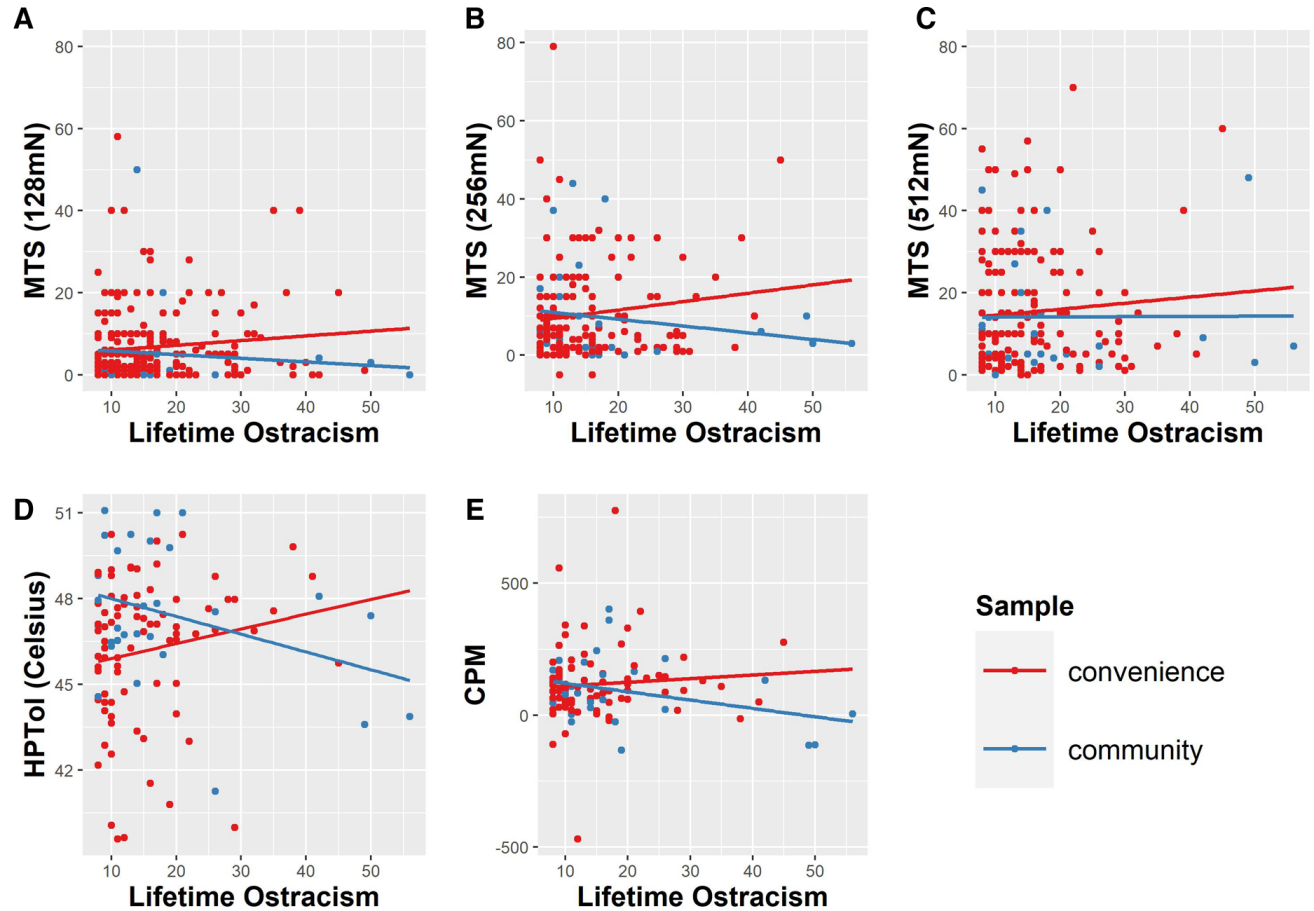
Sample population significantly (*p* < 0.05) moderated the relationship between lifetime ostracism experiences and (**D**) heat pain tolerance (HPTol^°C^) and (**B**) mechanical temporal summation (MTS) using the 256 mN weighted probe. A similar, yet non-significant pattern was observed for the relationship between lifetime ostracism and (**A** & **C**) MTS using other weighted probes as well as (**E**) conditioned pain modulation (CPM). The raw, untransformed values are presented in the figure.

Probing for study effects revealed a consistent pattern such that when study effects were present, they were always in the direction of the community study revealing a different pattern than the studies that included convenience sampling. Therefore, we report (*post-hoc*) moderation by sampling strategy (convenience sample vs. community sample) rather than individual study (Figure 2). There was a significant moderation effect of sample population on the relationship between lifetime ostracism experiences and HPTol^°C^ [ΔR^2^ = 0.050, *F*(1, 110) = 6.102, *p* = 0.015] and MTS at weight 256 mN [ΔR^2^ = 0.020, *F*(1, 195) = 4.055, *p* = 0.045]. Within group analyses show that lifetime ostracism experiences were associated with lower HPTol^°C^ among the community sample [*r*(33) = −0.348, *p* = 0.047], but were not associated with HPTol^°C^ among convenience samples [*r*(81) = 0.167, *p* = 0.136]. There were patterns of greater ostracism experiences to be associated with less MTS at weight 256 mN within the community sample [*r*(33) = −0.252, *p* = 0.158], but greater MTS within convenience samples [*r*(166) = 0.127, *p* = 0.102]; however these patterns were not statistically significant. While sample population was not a significant moderator for other laboratory pain measures, there were trends between lifetime ostracism experiences and CPM (*p* < 0.10). Greater lifetime ostracism experiences tended to be associated with less CPM within the community sample [*r*(33) = −0.312, *p* = 0.077], but not with CPM in convenience samples [*r*(81) = 0.089, *p* = 0.428]. Inspection of scatterplots suggests that across stimulus intensities, sample type was associated with opposite patterns of association between ostracism and MTS, such that the community sample showed patterns of slightly negative association between lifetime ostracism experiences and MTS, while the convenience sample showed slightly positive association.

#### 3.3.4. Racialized identity

**Figure 3:**
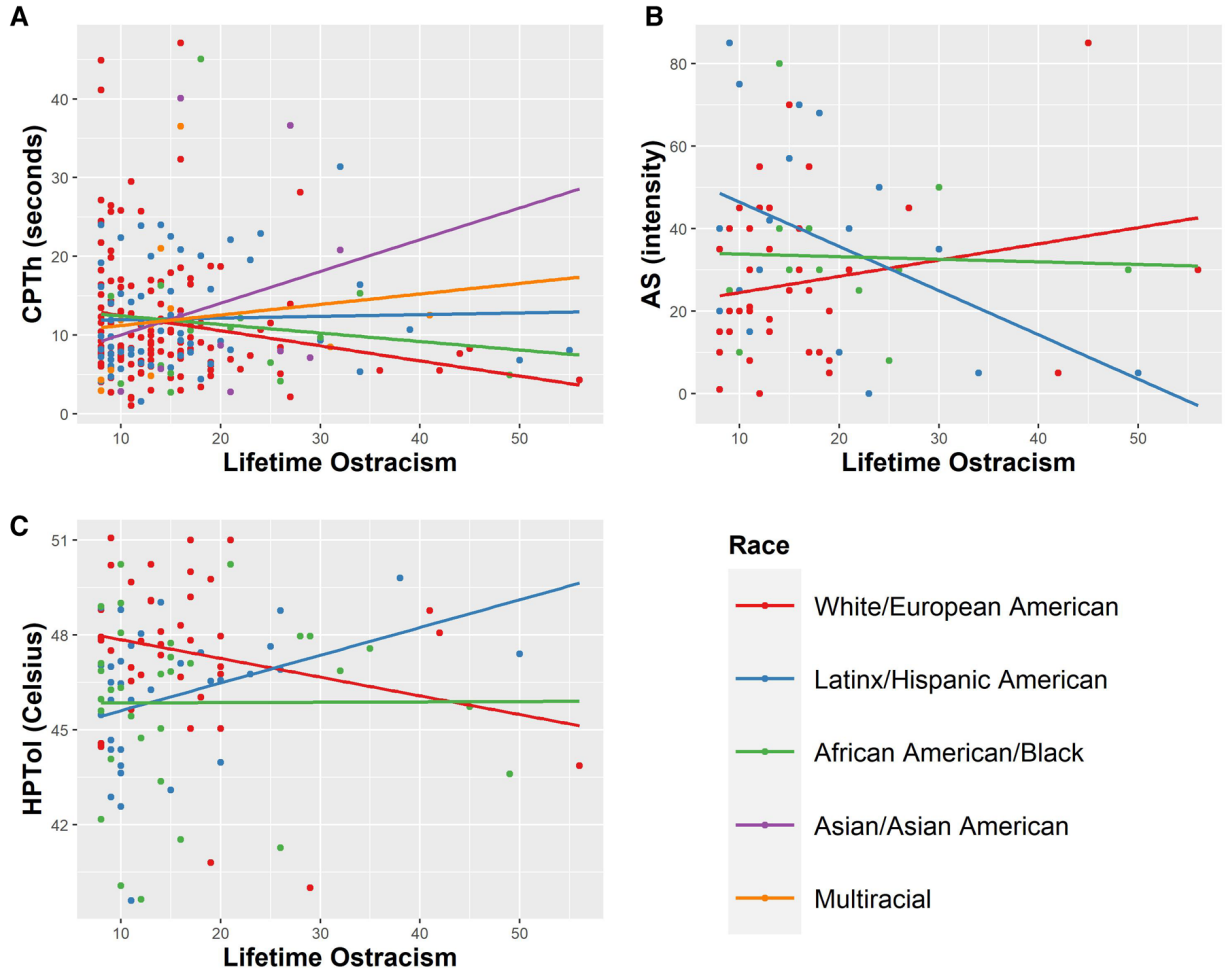
Racialized identity significantly (*p* < 0.05) moderated the relationship between (**A**) cold pain threshold (CPTh^sec^), (**B**) after sensations 30 s after cold stimulus cessation (**A**S^intensity^), and (**C**) heat pain tolerance (HPTol^°C^). The raw, untransformed values are presented in the figure.

Racialized identity significantly moderated the relationship between lifetime ostracism experiences and CPTh^sec^ [ΔR^2^ = 0.045, *F*(4, 222) = 2.65, *p* = 0.034], HPTol^°C^ [ΔR^2^ = 0.068, *F*(3, 103) = 2.75, *p* = 0.047], and AS^intensity^ [ΔR^2^ = 0.095, *F*(3, 74) = 2.76, *p* = 0.048] (Figure 3).

There was no main effect of racialized group on lifetime ostracism experiences [*F*(6, 500) = 1.153, *p* = 0.330]. However, there was a pattern of more lifetime experiences of ostracism among participants in racialized groups (participants identifying as Asian or Asian American: *M* = 18.11, *SD* = 8.45; those identifying as groups that weren't listed: *M* = 17.25, *SD* = 6.08; Black or African American: *M* = 16.95, *SD* = 9.75; Multiracial: *M* = 16.39, *SD* = 8.84; Native American or Alaskan Native: *M* = 16.25. *SD* = 14.57; Hispanic or Latinx American *M* = 15.63, *SD* = 8.69; White or European American: *M* = 14.85, *SD* = 7.64). Among participants that identified as White, greater ostracism experiences were associated with lower CPTh^sec^ [*r*(137) = −0.198, *p* = 0.020] and CPTh^°C^ [*r*(42) = 0.416, *p* = 0.006]. For Latinx participants, greater ostracism experiences were associated with lower CPInt^max^ [*r*(19) = −0.575, *p* = 0.010] and higher HPTol^°C^ [*r*(35) = 0.366, *p* = 0.031]. There was also a trend for greater lifetime ostracism experiences to be associated with lower AS^intensity^ [*r*(19) = −0.448, *p* = 0.055] within Latinx participants, but this trend did not reach statistical significance. Ostracism experiences were not associated with any laboratory pain measures for participants identifying as Asian or as Multiracial.

## 4. Discussion

In the current study, we demonstrate for the first time that greater lifetime experiences of ostracism are associated with laboratory pain, and specifically with lower CPTol^sec^. This is consistent with previous work that has demonstrated links between acute laboratory episodes of ostracism on cold pain (23). However, no other simple associations between lifetime ostracism and laboratory pain were observed. Indeed, while a strength of this study was the inclusion of dynamic measures, only the static measure of cold pain tolerance was significantly associated with lifetime ostracism experiences within whole group analyses. Although null findings for other measures may reflect several underling mechanisms (as discussed below), taken together with the extant literature, the current findings suggest it is possible that static measures of cold pain are the most sensitive to experiences of ostracism. Some studies have demonstrated a link between feelings of exclusion and cold sensations [e.g., experimental ostracism is associated with perceiving room temperatures as colder as well as with lower body temperatures (34, 35) drinking cold water in the laboratory was associated with lower levels of belongingness (36)]. Further work is needed to confirm whether static measures of cold pain are more sensitive to the variability of ostracism experiences.

Importantly, when sample diversity was considered as a relevant moderator, a more nuanced pattern of relationship between lifetime ostracism experiences and laboratory pain emerged. Specifically, planned analyses examining this relationship within racialized groups indicates that patterns between lifetime experiences of ostracism and acute pain sensitivity are obscured when racialized social oppression and privilege is not considered (i.e., when racialized identity is not considered in analyses). Notably, this is despite the lack of statistically significant differences in lifetime ostracism between racialized groups in this sample. Within participants that self-identified as White, greater lifetime ostracism experiences were associated with lower CPTh^sec^ and CPTh^°C^. Within Latinx participants, greater lifetime experiences of ostracism were associated with lower CPInt^max^ and higher HPTol^°C^. It is important to consider that there may be differential patterns in social indicators of pain among racialized groups—highlighting the importance of analyzing within groups to understand potential nuance in experiences of pain inequity. Furthermore, ostracism experiences of racialized groups are largely underrepresented within the literature. The current measures used to assess ostracism experiences may not fully capture ostracism experiences that are unique to racialized groups—who systematically experience greater ostracism across levels (e.g., interpersonally, structurally, culturally). In the current study, the differences in lifetime experiences of ostracism between racialized groups were not statistically significant. While the measure we used to assess lifetime experiences of ostracism has been validated within a racialized group (28, 29), it could also be missing important nuance in the ostracism experienced by different racialized groups or ostracism experiences occurring at different levels. More research is needed that examines the ostracism experiences of individuals within racialized groups, and how this may create or maintain pain inequities.

Further, our diversity approach revealed differential patterns of relationships between male and female participants. Lifetime experiences of ostracism were significantly associated with lower CPTh^sec^ and CPTol^sec^ only within male participants. We did not hypothesize differential patterns between male and female participants. However, this could point to potential differences between males and females in other factors that may mitigate the effects of ostracism such as social support and support seeking after experiences of ostracism. Social support is known to buffer the effects of life stressors on an individual's well-being and health including pain outcomes (37–40). Indeed, prior research suggests that receiving social support from a close other after being ostracized reduces negative emotions and brain activity associated with being excluded (41, 42). Although there is no evidence to suggest sex differences in responses to ostracism, there is evidence to support that men may be less likely to seek help or support from others when experiencing distress due to masculine gender roles of self-reliance and stoicism (43–46). Therefore, it is possible that sex differences in social support and support seeking may be an important factor that alters the relationship between ostracism experiences and pain sensitivity. Future research may consider how different responses to ostracism or other social modulators may influence the relationship between lifetime ostracism experiences and pain. Additionally, it is likely that stigmatized gender identities are associated with greater ostracism experiences and should be followed up in future work.

Importantly, *post-hoc* analyses revealed an unexpected pattern within sampling subgroups (convenience vs. community sample). Greater lifetime ostracism experiences were associated with lower HPTol^°C^ within the community sample but were not associated among the convenience sample. There were also trends for greater lifetime ostracism experiences to be associated with less MTS and CPM within the community sample compared to the convenience sample. A majority of the participants that completed the studies included in the current analysis were recruited on-campus using convenience sampling and were often students or other individuals affiliated with the university. Because of this, the studies that used convenience sampling consisted of primarily young, healthy, and college-educated adults who tend to report lower levels of ostracism. Our community sample, which excluded anyone affiliated with university, was relatively older and tended to report higher levels of lifetime ostracism experiences on average compared to the convenience sample. These trends may suggest a differential pattern among community samples who are likely to encounter more ostracism within their lifetime or perhaps more severe incidences of ostracism. This is important to consider because most of psychology research is conducted in undergraduate students using convenience sampling which limits its generalizability to other populations. Even within pain research, particularly research within non-clinical or chronic pain samples, a considerable amount is conducted with convenience samples with limited recruitment strategies and sampling within local communities. As a result, current models of social indicators of pain and pain inequity may be missing important nuance in the experiences of individuals that are often inadvertently excluded from participating in research.

There are several limitations that should be considered when interpreting the current findings. First, we report all comparisons within this exploratory study which increases the possibility for Type 1 error. Future research is needed to confirm and clarify the relationships between lifetime ostracism experiences and cold pain sensitivity. We also report non-significant results to spur future research and to support meta-analyses. However, these should be interpreted with caution. Second, due of differences in the studies sampled as well as the inclusion criteria for the present analysis, there were discrepancies in sample sizes across inferential analyses. This may impact the current findings, given that inferential analyses for certain laboratory pain measures had smaller sample sizes and less power to detect relationships. Third, most participants scored below the midpoint of the lifetime ostracism measure which could limit our ability to make inferences on those that experience moderate to high levels of ostracism. Previous research has shown that chronic pain is prevalent within groups that experience high levels of exclusion and marginalization such as homeless populations (47–49). However, further research is needed to explore the relationship between ostracism and pain sensitivity within individuals that have experienced moderate to high levels of ostracism. Fourth, while this study is novel in that it includes a measure of accumulated lifetime ostracism experiences, this measure is unable to assess the severity of ostracism experiences. Prior research suggests that the severity of ostracism experiences may show different patterns on acute pain sensitivity, in that severe experiences of ostracism may be associated with hyposensitivity to pain stimuli (26). This measure also does not assess ostracism experiences that are related to an individual's identity which is likely an important aspect of ostracism experienced by individuals with stigmatized or minoritized identities. In one study, Goodwin and colleagues (50) show that individuals that experienced ostracism related to their racialized identity had slower recovery of basic needs than individuals where ostracism was not related to racialized identity. Greater experiences of racialized exclusion or rejections have also shown to be associated with greater post-traumatic stress and depression symptoms (51). However, more research is needed to assess the severity of ostracism experiences, as well as the attributions of ostracism to examine how these variables may influence the pain experiences of minoritized communities. Fifth, while the experience of acute laboratory pain is clinically relevant (52), it is important for future studies to examine the relationship between real-lived experiences of ostracism among individuals with chronic pain. Though loneliness and social isolation are known to enhance pain in this population, it is also expected that people with chronic pain experience more overt experiences of ostracism due to the frequent stigmatization of people with chronic pain conditions—especially for pain conditions with no biological or specified cause (53–56). Future work is needed to evaluate the independent effects of ostracism in the context of chronic and persistent clinical pain. Lastly, it is important to note that there were many null findings in the current study. It is possible that some of these findings represent small effects, true null findings, or that there are differential patterns related to the diversity of lived experiences across groups. Our moderation results indicate that differential patterns among groups are at least partially present.

Traditional approaches to understanding pain disparities typically control for demographic factors and identities that confound pain experiences due to social indicators. This approach tells us little about the reasons as to why and how pain inequities exist. By controlling for diverse identities, this approach discards the meaningful variance in experiences of social inequity (the root cause of pain disparities) among racialized and other marginalized identities that influence pain physiology. In the current study, we provide a new potential approach that retains and explores the diversity of our sample to understand one potential mechanism—lifetime ostracism experiences—by which social inequity may contribute to pain inequities within marginalized groups. Instead of using diverse identities as control variables, we conducted within-group analyses to examine whether there were different patterns between lifetime ostracism experiences and acute sensitivity to laboratory pain across age and within sex and racialized groups. Future work is needed to confirm and extend the current findings by including and representing non-convenience populations—particularly groups that experience greater levels of ostracism or marginalization to assess patterns on lived pain experiences. As noted above, future research in this area may specifically seek to (1) confirm the specificity of the relationship between ostracism and cold-pain and further probe mechanistic explanations for this relationship, (2) examine specific racialized ostracism experiences and associations with pain using focused within-group approaches, (3) examine the ostracism-pain relationship among stigmatized gender groups, and (4) expand the development and validation of social indicators of pain using non-convenience samples. Additional research is also needed to assess other dimensions of social injustice across levels (e.g., interpersonal, structural, cultural) and how they create and maintain pain inequities (15).

## Data Availability

The raw data supporting the conclusions of this article will be made available by the authors, without undue reservation.
